# Supplementary figures and images for: Determination of Shear Bond Strength between PEEK Composites and Veneering Composites for the Production of Dental Restorations
Source: Materials (Basel). 2023 Apr 22;16(9):3286. doi: 10.3390/ma16093286 (PMC10178894; doi:10.3390/ma16093286)

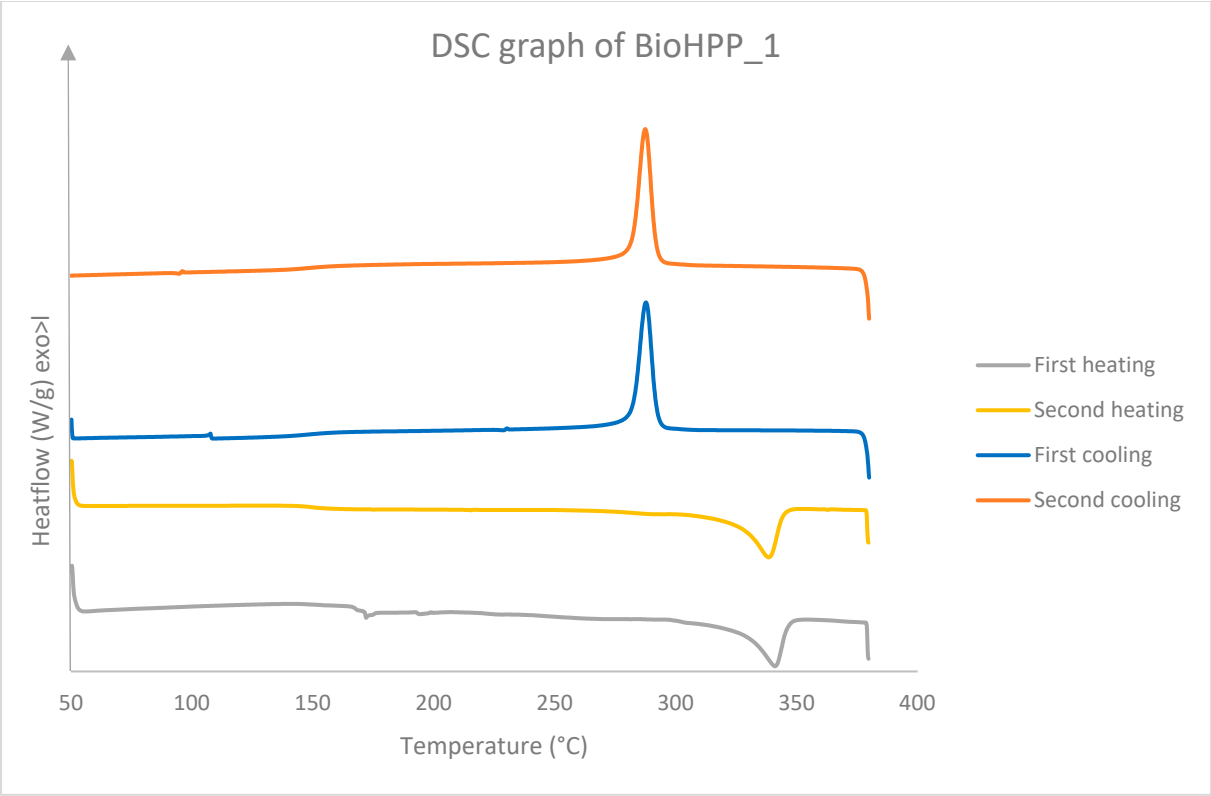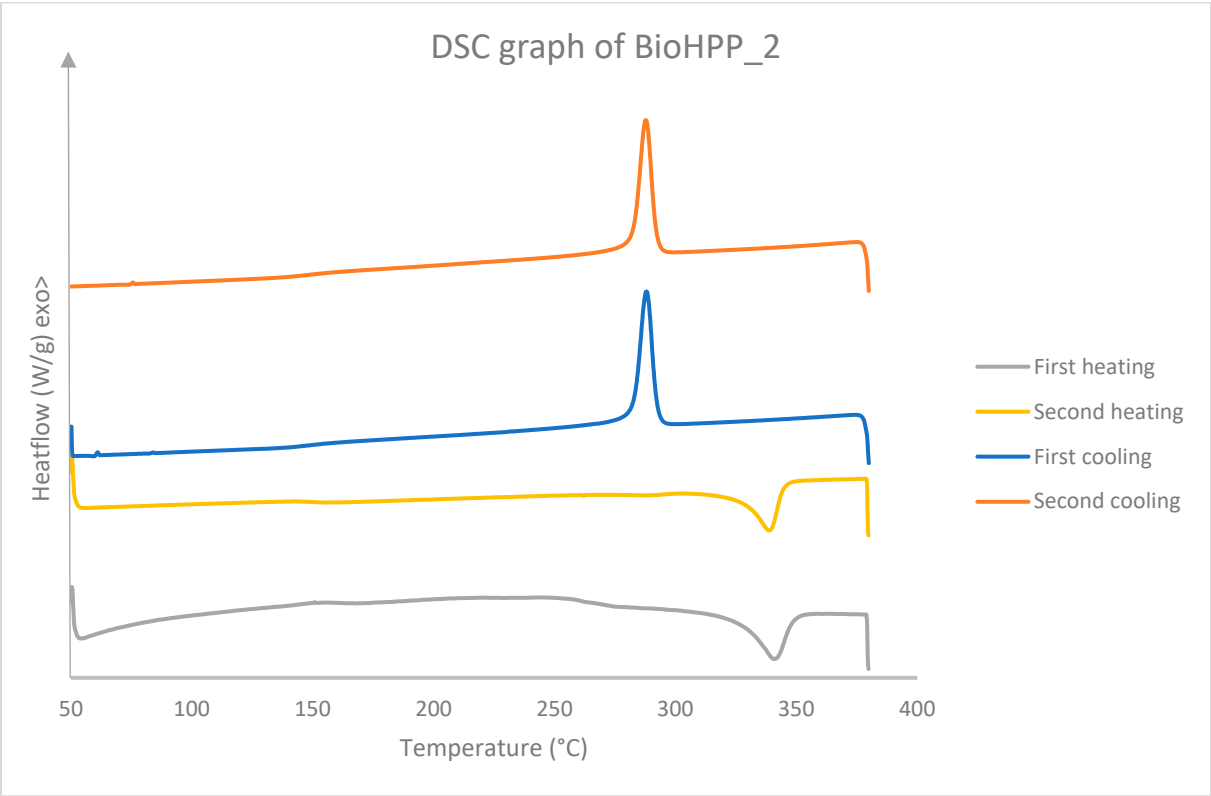

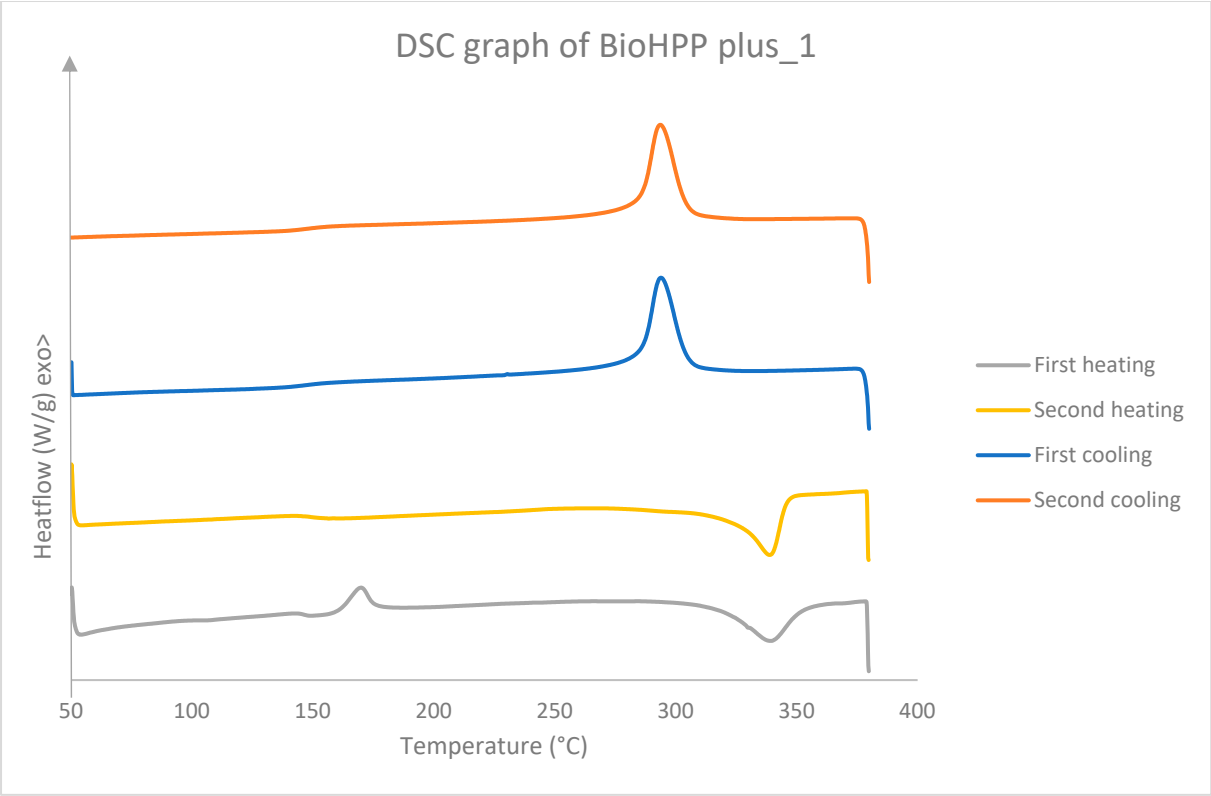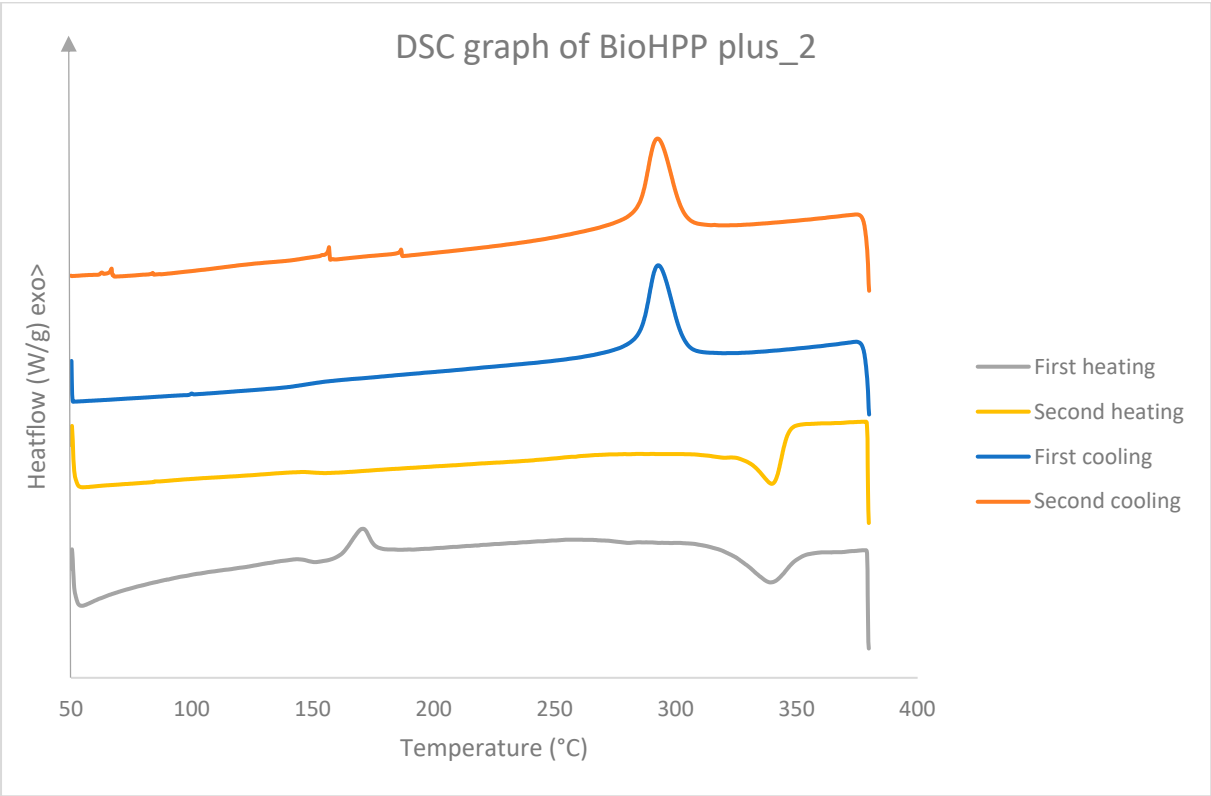

Supplement: Supplementary file 1 [file materials-16-03286-s001.zip › materials-2326740-supplementary.pdf]
